# Nucleus basalis functional connectivity aberrations in temporal lobe epilepsy and improvements after successful epilepsy surgery

**DOI:** 10.1002/epi.70451

**Published:** 2026-08-19

**Authors:** Addison C. Cavender, Derek J. Doss, Ghassan S. Makhoul, Bruno Hidalgo Monroy Lerma, Anas Reda, C. Price Withers, Emily Liao, Kate Wang, Hernán F. J. González, Sarah E. Goodale, Graham W. Johnson, Laurie E. Cutting, Sarah K. Bick, Catie Chang, Victoria L. Morgan, Dario J. Englot

**Affiliations:** 1 Vanderbilt Brain Institute, Vanderbilt University, Nashville, Tennessee, USA; 2 Vanderbilt University Institute of Imaging Science, Vanderbilt University, Nashville, Tennessee, USA; 3 Vanderbilt Institute for Surgery and Engineering, Vanderbilt University, Nashville, Tennessee, USA; 4 Department of Biomedical Engineering, Vanderbilt University, Nashville, Tennessee, USA; 5 Department of Neurological Surgery, Vanderbilt University Medical Center, Nashville, Tennessee, USA; 6 Department of Neurological Surgery, University of California, San Diego, La Jolla, California, USA; 7 Department of Neurologic Surgery, Mayo Clinic, Rochester, Minnesota, USA; 8 Department of Special Education, Vanderbilt University, Nashville, Tennessee, USA; 9 Department of Electrical and Computer Engineering, Vanderbilt University, Nashville, Tennessee, USA; 10 Department of Neurology, Vanderbilt University Medical Center, Nashville, Tennessee, USA; 11 Department of Radiology and Radiological Sciences, Vanderbilt University Medical Center, Nashville, Tennessee, USA

**Keywords:** brain networks, functional neuroimaging, MRI, seizures

## Abstract

**Objective:**

Resective surgery achieves seizure freedom in approximately 60%–80% of patients with drug-resistant temporal lobe epilepsy (TLE), yet the extent to which seizure cessation permits recovery of disrupted functional brain networks remains unclear. Increasing evidence suggests that recurrent seizures alter subcortical–cortical communication involved in arousal and cognition, contributing to chronic interictal deficits, such as in executive function. Prior work demonstrates that the nucleus basalis of Meynert (NBM), the primary source of cortical cholinergic input to the cortex, exhibits aberrant functional connectivity in patients with TLE. We examined NBM connectivity in patients with TLE before and after surgery using patient-specific NBM segmentations. We sought to investigate whether seizure freedom is associated with recovery of NBM functional connectivity, consistent with previous observations of interictal network dysfunction reversal in other subcortical arousal structures.

**Methods:**

Resting-state functional magnetic resonance imaging was analyzed in 75 patients with TLE, including 34 patients postoperatively, and 106 controls. Patient-specific NBM segmentations were used to compute functional connectivity between the NBM and frontoparietal association cortex (FPAC) and across the whole brain. Subcortical arousal network community structure was assessed using community detection. Postoperative network changes were evaluated relative to seizure outcome.

**Results:**

Prior to surgery, patients exhibited reduced bilateral NBM connectivity to the FPAC and whole brain compared to controls, indicating widespread cholinergic network disruption beyond the ipsilateral hemisphere. Following surgery, NBM connectivity increased and no longer differed from controls. Seizure-free patients demonstrated greater postoperative increases in ipsilateral NBM-FPAC connectivity than non-seizure-free patients, which was not explained by time to postoperative scan or volumetric change. Subcortical arousal community structure remained altered postoperatively.

**Significance:**

Successful TLE surgery is associated with partial restoration of cholinergic arousal network connectivity, supporting the idea that seizure cessation enables recovery of brain networks disrupted by recurrent seizures. These findings position the NBM as a potential biomarker of network recovery following epilepsy surgery.

## 1 | INTRODUCTION

Epilepsy affects 1% of the population, and temporal lobe epilepsy (TLE) is the most common form of focal epilepsy.^1^ Patients with TLE suffer from debilitating seizures that disrupt large-scale functional brain networks specifically associated with arousal. Additionally, many patients suffer from cognitive deficits associated with disrupted subcortical arousal systems, such as executive functioning and attention.^2^ The Extended Network Inhibition Hypothesis (ENIH) proposes that recurrent focal seizures lead to widespread disruption of subcortical–cortical connectivity networks that support arousal and cognitive functioning.^3^ Supporting this framework, prior work has shown that patients with TLE exhibit reduced functional connectivity (FC) of key subcortical arousal systems, including the ascending reticular activating system and thalamus.^4–6^ Reduced FC of subcortical arousal systems is associated with increased seizure burden and more severe interictal neurocognitive symptoms, particularly in memory and language deficits.^4,5,7^ However, these arousal network disturbances may not be permanent. Thalamic and brainstem arousal network connectivity has been shown to recover following epilepsy surgery, particularly in patients who achieve seizure freedom.^5,6^ This suggests that seizure cessation may permit functional reorganization or normalization of disrupted subcortical–cortical circuits, implying that arousal network abnormalities in TLE may be driven by ongoing seizures.^8^

The nucleus basalis of Meynert (NBM), located in the basal forebrain, is highly implicated in arousal.^9^ The NBM provides direct cholinergic projections to widespread cortical regions, including the frontoparietal association cortex (FPAC), insula, temporal cortex, and limbic structures.^10^ Furthermore, anatomical and stimulation studies demonstrate that the cortical regions the NBM innervates are critical for sustained attention, cognitive control, sensory integration, and consciousness, positioning the NBM as a central modulator of cortical arousal.^9,10^ Across species and neurological disorders, disruption of NBM cholinergic signaling is consistently linked to reduced cortical activation, impaired vigilance, and cognitive decline.^11–13^ Lesions to the NBM result in increased cortical slow-wave activity (a signature of low arousal),^9^ and degeneration of basal forebrain cholinergic nuclei is strongly associated with attentional and memory deficits in neurodegenerative disorders such as Alzheimer's disease, Parkinson's disease dementia, and dementia with Lewy bodies.^13^ Recent studies have shown that the NBM might be a potential target for neuromodulation to improve arousal deficits associated with these diseases.^14,15^

The NBM has also been associated with network dysfunction in patients with TLE. Patients with TLE exhibit reduced FC between the basal forebrain and distributed cortical networks, particularly the FPAC.^16^ Previous findings suggest alterations of subcortical arousal structure connectivity to the cortex are most pronounced in the FPAC, and critically, the FPAC is also the area during seizures that is most affected by depressed activity and an increase in slow waves.^5,6,17^ Thus, we decided to investigate the FPAC as an a priori area of interest. Reduced FPAC connectivity has been associated with greater seizure burden, and FPAC activity is essential for maintaining arousal, sustained attention, cognitive control, and consciousness.^18^ Seizure propagation in TLE may suppress FPAC activity, contributing to impaired consciousness during seizures and reduced interictal vigilance.^17,19^ Consistent with the ENIH, recurrent seizures may therefore drive progressive disruption of NBM-FPAC connectivity, reinforcing pathological network states during the interictal period.^3^ NBM connectivity has also been associated with measures of attention,^20^ suggesting that cholinergic basal forebrain dysfunction due to recurrent seizures may contribute to impaired vigilance in epilepsy.

Seizures have been shown to suppress cholinergic neurons in both the basal forebrain and brainstem, leading to decreased cortical cholinergic transmission and impaired consciousness during seizures.^12^ These findings provide a link between recurrent seizures and sustained dysfunction of cholinergic arousal systems, reinforcing the NBM as a plausible mediator of network-level cognitive and arousal deficits in TLE. However, it remains unclear whether NBM dysfunction represents a primary contributor to arousal impairment or a downstream consequence of recurrent seizures.

Critically, approximately 30%–40% of patients continue to have seizures despite trials of at least two antiseizure medications. For these individuals, epilepsy surgery is the most effective treatment option, with approximately 60%–80%^21,22^ achieving seizure freedom following resection. Although historically considered a focal disorder, TLE is increasingly recognized as a network disease characterized by widespread disruptions extending beyond the seizure onset zone. Resective epilepsy surgery induces widespread changes in FC,^23^ including in regions distal to the site of resection,^24^ thus it is understood that surgical intervention perturbs global brain networks rather than solely local epileptogenic tissue. As mentioned previously, preoperative network disturbances are associated with interictal neurocognitive symptoms,^2^ underscoring the clinical importance of understanding large-scale network organization in these patients. Longitudinal imaging studies demonstrate that postoperative functional changes are strongest in regions structurally connected to the resected temporal lobe and may reflect damage, recovery, or other reorganization.^25^ If NBM connectivity abnormalities recover following successful surgery, but not when seizures persist, this would support a reversible seizure-driven network disturbance. Conversely, persistent abnormalities would suggest more durable cholinergic network disruption, potentially one that has not recovered by the time of observation and may contribute to ongoing interictal cognitive dysfunction. Additionally, these changes in the absence of volumetric changes support the notion that network changes are due to FC differences, rather than structural disturbances caused by surgery. Despite recognition of the NBM as a key arousal hub and its role in TLE, postoperative FC of the NBM following epilepsy surgery has not been examined. Thus, it remains unclear whether NBM network abnormalities persist after surgery or whether successful seizure control permits recovery of cholinergic arousal circuits previously disrupted by recurrent seizures, potentially enabling improvement in interictal neurocognitive deficits.

Although pairwise FC quantifies the strength of coupling between two regions or two groups of regions, network-level measures such as community detection characterize higher scale network architecture by identifying groups of regions that are more strongly functionally connected to each other than to the rest of the brain and thus are thought to share similar qualities. This approach enables assessment of the functional organization of the subcortical arousal system, allowing investigation of its potential reorganization in TLE and potential changes in network topology following surgery. By examining community structure in addition to pairwise FC across controls, preoperative patients, and postoperative patients, we can begin to assess whether epilepsy surgery reshapes large-scale integration of the NBM within the broader arousal system.

In the present study, we examine NBM FC changes after epilepsy surgery in patients with TLE, extending our previous work demonstrating altered NBM connectivity in TLE prior to surgery.^16^ To our knowledge, this is the first investigation of postoperative NBM connectivity. The present study additionally uses a novel patient-specific NBM segmentation approach and utilizes an increased cohort size. By leveraging novel machine-learning patient-specific NBM segmentations^26^ and longitudinal postoperative imaging, we aimed to determine whether successful seizure control is associated with recovery of disrupted cholinergic arousal networks. We hypothesized that patients who achieve seizure freedom would demonstrate postoperative increases in NBM FC, particularly in the hemisphere ipsilateral to the seizure focus, toward levels observed in healthy controls.

## 2 | MATERIALS AND METHODS

### 2.1 | Study participants

Participants consisted of 75 patients with unilateral TLE who underwent epilepsy surgery at Vanderbilt University Medical Center between 2012 to 2023 (Table 1). Diagnosis of TLE was established according to standard presurgical epilepsy evaluation, including detailed clinical history, structural magnetic resonance imaging (MRI), inpatient video electroencephalography, positron emission tomography, functional MRI or Wada testing for eloquent cortex localization, and comprehensive neuropsychological assessment. All surgical cases were discussed at a multidisciplinary epilepsy conference including neurologists, neurosurgeons, neuropsychologists, radiologists, and other physicians. Of the 34 postoperative patients, the majority (*n* = 24) underwent a selective amygdalohippocampectomy (SAH; Table 1). Our SAH approach has previously been described and is standard across patients at our center.^27^ Repeat resting-state functional MRI (fMRI) was acquired in 34 patients with unilateral TLE following surgery (mean ± SD = 30.3 ± 15.1 months). Postoperative seizure outcome was determined by epileptologist evaluation of patient and family reports at the timepoint of follow-up scan using the Engel outcome score.^28^ Here, seizure freedom is defined as Engel IA (*n* = 18); all other outcomes were classified as non-seizure-free (*n* = 16). A comparison group of 106 healthy adult controls was also included (Table 1). All participants provided written informed consent for participation in the study. All procedures were approved by the Vanderbilt University Institutional Review Board. Additionally, we compared clinical variables between patient and control groups, between seizure-free and non-seizure-free patient cohorts (Table S1), and between preoperative and postoperative patient cohorts (Table S2). No groups differed significantly on any clinical variables of interest, including age, sex, handedness, duration, seizure frequency, history of focal-to-bilateral tonic–clonic seizures, side of seizure onset, and number of antiseizure medications.

### 2.2 | Imaging

In concordance with our previous studies,^16^ data were obtained using a 3-T MRI scanner (Philips Achieva, Philips Healthcare) with a 32-channel head coil. Each MRI session consisted of (1) three-dimensional (3D) T1-weighted whole-brain images for tissue segmentation (gradient echo, repetition time [TR] = 9.10 ms, echo time [TE] = 4.60 ms, 192 shots, flip angle = 8.0°, matrix = 256 × 256, 1.0 mm^3^); (2) 2D T1-weighted axial scan acquired in the same slice orientation as the functional images for coregistration of functional to structural images (1.0 mm × 1.0 mm × 3.5 mm with .5-mm gap); and (3) two consecutive 10-min eyes-closed resting-state T2*-weighted blood oxygenation level-dependent (BOLD) fMRI scans (field of view = 240.0 mm, TE = 35.0 ms, TR = 2.0 s, 34 axial slices, slice thickness = 3.50 mm with a .50-mm gap, matrix = 80 × 80, 3.0 × 3.0 × 4.0 mm^3^), with 300 volumes acquired during each resting-state acquisition. All participants were instructed to lie at rest with their eyes closed for the entire resting-state fMRI session. We recorded respiratory and cardiac fluctuations at 500 Hz using the Phillips scanner system.

### 2.3 | Regions of interest

Regions of interest (ROIs) were selected to capture cortical, subcortical, and brainstem components of arousal networks previously implicated in TLE. In total, 111 ROIs were defined for FC analyses. These included 82 cortical ROIs from the Desikan–Killiany atlas using FreeSurfer (https://surfer.nmr.mgh.harvard.edu/fswiki/CorticalParcellation). From a subset of Desikan–Killiany atlas regions, we defined a group of frontal, parietal, and insular regions that previously demonstrated reduced connectivity with arousal structures in TLE.^29^ We additionally generated patient-specific thalamic segmentations, with 14 thalamic nuclei defined per participant.^30^ Thirteen brainstem nuclei were included from the Harvard Ascending Arousal Network Atlas.^31^ Patient-specific segmentations of the NBM were generated using a deep learning-based segmentation approach developed in our laboratory.^26^ A complete list of ROIs included in the patient-specific atlas is listed in Table S3. All nonmidline ROIs were designated as either ipsilateral or contralateral to the side of seizure onset for each patient. To standardize seizure onset laterality across participants, patient parcellations were flipped such that the ipsilateral hemisphere was aligned to the left side of the image. The same proportion of control scans were flipped to maintain hemispheric symmetry at the group level.

### 2.4 | Preprocessing

fMRI data were preprocessed with MATLAB 2024a (MathWorks) and SPM8. Preprocessing steps included slice-timing correction, motion correction, physiological noise correction using RETROICOR, and tissue segmentation (white matter, gray matter, cerebrospinal fluid). Data were temporally bandpass-filtered between .0067 and .1 Hz. All statistical analyses were conducted in patient-specific space.

### 2.5 | FC analyses

We examined undirected FC between the ipsilateral and contralateral NBM and the FPAC, as well as connectivity with the whole-brain atlas (excluding self-connections). For each 10-minute fMRI acquisition per participant, we computed FC between the ipsilateral and contralateral NBM and FPAC regions, as well as the whole brain (all ROIs in the atlas). Partial Pearson correlation coefficients were calculated between the mean BOLD time series of each region, generating a 111 × 111 FC matrix for each session per participant. Correlation matrices were Fisher *z*-transformed, and connectivity values were averaged across the two resting-state runs to produce a single matrix per participant. Comparisons of FC between patients and controls were completed using independent samples *t*-tests, and between preoperative and postoperative patients using paired samples *t*-tests. Statistical significance was defined a priori at *p* < .05. Post hoc correction for multiple comparisons was performed using Benjamini–Hochberg false-discovery rate (FDR).

To visualize connectivity differences between patients and controls, seed-to-voxel FC maps were computed for each participant using Fisher *z*-transformed Pearson correlations. FC maps were generated using ipsilateral, contralateral, and bilateral NBM seeds for each patient. A mean echo-planar imaging reference image was derived from each participant's resting-state scan and registered to the participant's T1-weighted anatomical image using rigid-body linear registration followed by boundary-based registration. This transformation was then applied to the participant's connectivity *z*-map to bring it into anatomical space. The participant's T1-weighted image was linearly registered to the MNI152 template using an affine transformation, followed by nonlinear registration. The resulting nonlinear warp field was applied to the participant's connectivity z-map to transform it into standard MNI space. *Z*-maps were spatially smoothed with a 4-mm full-width half-maximum Gaussian kernel. MNI-space *z*-maps were entered into group analyses using FSĽs *randomise*.^32^ Statistical inference was performed using permutation testing with threshold-free cluster enhancement^33^ using FSĽs *randomise* (5000 permutations). Voxels surviving familywise error (FWE) correction (*p*_FWE_ ≤ .05) were projected onto an inflated cortical surface for visualization.

We additionally used a generalized linear model to examine the relationship between postoperative FC and seizure outcome, accounting for potential confounding variables. Change in FC (postoperative–preoperative ipsilateral NBM to FPAC FC) was treated as the dependent variable, with seizure outcome (seizure-free vs. non-seizure-free) as the primary predictor of interest, and change in NBM volume (postoperative–preoperative ipsilateral NBM volume) and time from surgery to latest postoperative scan included as covariates (FC ~ outcome + volume + time).

### 2.6 | Community detection

Community detection was applied to determine whether epilepsy surgery influences higher order arousal network organization that is not captured by pairwise connectivity measures. Thus, we examined community organization in patients and controls to identify groups of brain regions exhibiting stronger within-community than between-community connectivity. We applied the Louvain community detection algorithm to each individual's connectivity matrix with a resolution parameter sweep (*γ* = 1.3–2.5, step = .01), maximizing modularity at the individual level. For each *γ*, a group-level consensus partition was derived by constructing a matrix reflecting the proportion of participants assigning pairs of regions to the same community. Partition stability across *γ* values was quantified using normalized mutual information between group-level consensus partitions, and *γ* = 2.0 was selected based on maximal stability for defining group-level community structure. Community assignments of subcortical arousal ROIs were evaluated in healthy controls, preoperative patients, and postoperative patients as a qualitative topological observation. Specifically, changes in community assignment would indicate reorganization of network topology (altered patterns of functional segregation and integration), whereas stability of community structure despite pairwise connectivity changes would suggest preservation of higher order network organization.

### 2.7 | Volume comparison

Ipsilateral and contralateral NBM volumes (mm^3^) of patients were extracted from subject-specific NBM segmentations to assess whether surgery altered NBM volume. Analyses were restricted to patients with both preoperative and postoperative scans. NBM volumes were also extracted from subject-specific segmentations of healthy controls. As stated previously, to standardize epileptogenic hemisphere laterality across patients, patient parcellations were flipped such that the ipsilateral hemisphere was aligned with the left side of the image. The same proportion of control scans were flipped to maintain hemispheric symmetry across the whole group. Preoperative and postoperative patients were compared using paired samples *t*-tests, whereas patient groups and controls were compared using independent samples *t*-tests.

## 3 | RESULTS

### 3.1 | NBM shows reduced connectivity in patients with TLE preoperatively compared to healthy controls

Relative to controls, preoperative patients with TLE exhibited reduced FC between bilateral NBM and FPAC, as well as with the whole brain. Specifically, connectivity was reduced for ipsilateral NBM-FPAC (Figure 1A; *p*_FDR_ = 1.4 × 10^−3^, independent samples *t*-test), ipsilateral NBM–whole brain (Figure 1B; *p*_FDR_ = 1.3 × 10^−3^), contralateral NBM-FPAC (Figure 1C; *p*_FDR_ = 1.2 × 10^−2^), and contralateral NBM–whole brain (Figure 1D; *p*_FDR_ = 3.0 × 10^−2^). Additionally, voxelwise comparison of NBM connectivity demonstrated that patients exhibited relatively diffuse reductions in NBM FC to the same hemisphere as the NBM compared to controls (Figure 1E,F).

### 3.2 | Patients show increased FC of the ipsilateral NBM following surgery

Following surgery, patients exhibited increased FC of the ipsilateral NBM, including increased connectivity with the FPAC (Figure 2A; *p*_FDR_ = 1.8 × 10^−2^, paired samples *t*-test) and the whole brain (Figure 2B; *p*_FDR_ = 8.4 × 10^−3^). Postoperative increases were also observed in the contralateral NBM, with greater connectivity to the FPAC (*p*_FDR_ = 8.0 × 10^−3^) and the whole brain (*p*_FDR_ = 1.0 × 10^−2^). Additionally, separate voxelwise comparisons of bilateral NBM connectivity in preoperative patients and postoperative patients compared to controls demonstrated that prior to surgery, patients show dispersed reductions in NBM FC (Figure 2D). However, following surgery, patients no longer exhibited bilateral NBM connectivity statistically different from controls.

### 3.3 | Seizure-free patients show greater postoperative increases in ipsilateral NBM-FPAC connectivity than non-seizure-free patients

To determine whether postoperative connectivity changes were associated with seizure outcome, we compared seizure-free and non-seizure-free patients. Seventy-eight percent of seizure-free patients showed an increase in FC following surgery compared to 62.5% of non-seizure-free patients. Seizure-free patients exhibited greater increases in ipsilateral NBM-FPAC FC than patients with continued seizures (Figure 3A; *p*_FDR_ = 1.9 × 10^−2^, independent samples *t*-test). However, change in ipsilateral NBM-whole brain connectivity did not differ between seizure-free and non-seizure-free patients (Figure 3B; *p*_FDR_ = 1.2 × 10^−1^). A two-tailed one-sample *t*-test revealed that seizure-free patients showed a significant change in ipsilateral NBM-FPAC connectivity from pre- to postoperative scans (Figure 3A; *p*_FDR_ = 2.4 × 10^−2^), whereas non-seizure-free patients did not.

### 3.4 | FC differences following surgery were not explained by structural differences

As previous work has demonstrated subcortical atrophy in TLE, we examined NBM volume to determine whether postoperative functional changes reflected underlying structural differences. No differences in ipsilateral or contralateral NBM volume were observed between preoperative and postoperative patients. Interestingly, ipsilateral NBM volume was lower in patients prior to surgery compared to healthy controls (Figure 2C; *p*_FDR_ = 4.5 × 10^−3^, independent samples *t*-test). However, contralateral NBM volume was not reduced in patients prior to surgery compared to controls (*p*_FDR_ = 3.0 × 10^−1^). Ipsilateral NBM volume in postoperative patients was still significantly lower than healthy controls (Figure 2C; *p*_FDR_ < 1.0 × 10^−3^). In a generalized linear model with seizure outcome, NBM volume change (postop–preop), and time to postoperative scan as predictors, seizure outcome was the only significant predictor of change in FC (*β*= − 2.39, *p* = .010); NBM volume (*p* = .26) and time to postoperative scan (*p* = .86) did not reach significance.

### 3.5 | Patients show disrupted subcortical community structure compared to controls that does not reorganize following surgery

Community detection revealed marked differences in subcortical network organization between groups. In controls, 13 communities were identified, with the NBM forming a distinct community (Figure 4). In contrast, patients exhibited a collapse of subcortical community structure, with only four communities detected and subcortical regions clustering together. This altered community structure persisted after surgery, indicating that subcortical community structure did not normalize postoperatively. The figure conveys only the subcortical structures of interest.

## 4 | DISCUSSION

The present study is the first to our knowledge that examines postoperative connectivity of the NBM in patients with TLE. We demonstrate that NBM dysfunction in TLE includes both preoperative bilateral network disruption and postoperative outcome-dependent reorganization. Before surgery, patients with TLE exhibited reduced bilateral NBM FC relative to healthy controls, indicating that cholinergic arousal network disruption in TLE is bilateral and extends beyond previously reported unilateral network abnormalities. The reduced FC of the NBM in patients with TLE corroborates previous findings,^16^ even when accounting for patient-specific NBM segmentations and a larger cohort size. Following surgery, these connectivity differences were no longer present, suggesting recovery of NBM network organization. Whole-brain connectivity of the NBM increased across the full postoperative cohort, reflecting a general effect of surgery. Although NBM-FPAC FC also increased across the postoperative cohort, patients who achieved seizure freedom showed significantly greater postoperative increases in NBM-FPAC FC compared to non-seizure-free patients. Although the majority of patients across seizure-free and non-seizure-free cohorts demonstrated an increase in FC, the change in NBM-FPAC FC was greater in seizure-free patients, which may reflect stronger increase in FC due to seizure cessation. However, qualitative topological comparison indicated that broader subcortical community structure remained different from controls postoperatively, indicating that certain organizational features of the subcortical arousal system may be resistant to change following surgery. Thus, we demonstrate novel evidence that NBM FC abnormalities in TLE are modulated by seizure outcome following epilepsy surgery, and that NBM dysfunction in TLE includes both reversible and persistent features of cholinergic arousal network dysfunction. It is also worth noting that epilepsy surgery is not considered palliative.^34^ Non-seizure-free (non-Engel IA) patients may still experience clinically meaningful reductions in seizure burden, which may help explain connectivity increases observed even outside of the seizure-free (Engel IA) cohort.

The recovery of NBM-FPAC connectivity in seizure-free patients suggests that seizure cessation is associated with reorganization or normalization of connectivity. This builds upon the ENIH by demonstrating that interictal FC disturbances associated with recurrent seizures may be reversible. These results are consistent with the idea that ongoing seizures contribute to and maintain pathological network states, and that seizure cessation permits functional reorganization of disrupted subcortical–cortical circuits. Importantly, the absence of postoperative changes in NBM volume suggests that connectivity recovery reflects network-level functional reorganization rather than structural change within the subsequent postoperative years, aligning with broader models of postsurgical reorganization.^5,6,24^ Additionally, the seizure-free and non-seizure-free cohorts did not differ in epilepsy duration, monthly seizure frequency, history of focal-to-bilateral tonic–clonic seizures, side of seizures, pathology, time to postoperative scan, type of surgery, nor number of antiseizure medications (Table S1). Thus, although it is important to note that seizure activity is one plausible contributor to FC changes among several, our seizure-free and non-seizure-free cohorts did not differ in disease or clinical characteristics of interest, and thus we are positing that seizure cessation is contributing to these effects.

Although NBM-FPAC connectivity may have changed postoperatively, altered NBM–subcortical network organization in patients with TLE remained stable postoperatively. The finding that potentially dysregulated broader subcortical community structure remains stable postoperatively may suggest that recurrent seizures promote a persistently reorganized subcortical network in which the NBM is more functionally integrated with other arousal-related subcortical structures, including ascending reticular activating system structures. Such increased integration due to recurrent seizures may reflect a shift toward reduced functional differentiation within subcortical arousal networks, which could facilitate widespread propagation of seizure activity and further contribute to interictal impairments in attention and arousal. Longer term follow-up scans may be necessary to examine a reorganization of this altered subcortical network, if it occurs.

These findings may serve as groundwork for future clinical applications. Because neuromodulation acts on distributed brain networks rather than focal pathology,^35^ FC metrics may offer a useful framework for understanding how stimulation may restore or reshape pathological network interactions underlying seizures and impaired arousal. NBM connectivity measures may therefore serve as a candidate biomarker of recovery and seizure outcome, reflecting a shift toward more adaptive, seizure-free network states. Demonstrating normalization of NBM connectivity with seizure freedom therefore provides mechanistic support for targeting cholinergic arousal systems in future neuromodulation strategies, similar to approaches that have been explored in Parkinson disease and Lewy body dementia.^14^ By leveraging an expanded cohort and patient-specific NBM segmentations, this study was powered to detect bilateral effects of NBM connectivity that had not previously been detected.

There are limitations to this study that could be addressed in the future. The postoperative sample was smaller than the total presurgical cohort, and surgical approaches varied across patients. Future studies with larger available postoperative cohorts should consider stratifying by surgery type. We also did not account for medication changes following surgery, which may have influenced postoperative network reorganization independent of seizure control. Additionally, evaluation of postoperative seizure status relied on patient and family reports following surgery. Notably, this study did not examine associations between pre- to postoperative NBM FC and neuropsychological outcomes, as our current 1-year follow-up neuropsychological testing is likely too short to capture meaningful cognitive changes, which prior literature suggests may not be meaningful until 3–5 years postoperatively.^36^ Finally, static resting-state FC measures reflect time-averaged coupling and do not capture the state-dependent or epoch-dependent dynamics of subcortical–cortical communication.^37^ Future studies could consider explicitly modeling or monitoring arousal state throughout scanning to more precisely characterize the contribution of NBM connectivity to arousal across preoperative and postoperative patients with TLE and controls.

## 5 | CONCLUSIONS

Our findings indicate that NBM connectivity abnormalities in TLE may reflect seizure-related network disturbances that can partially recover following seizure cessation. The unique restoration of NBM-cortical connectivity in seizure-free patients provides support for a model in which recurrent seizures actively disrupt cholinergic arousal networks, and seizure control permits functional reorganization. These results position the NBM as a dynamic hub within arousal networks and highlight its potential value as a biomarker of network recovery and treatment response. Overall, this work provides a mechanistic foundation for future studies targeting cholinergic arousal systems in epilepsy and exploring the NBM as a candidate site for neuromodulation.

## Supplementary Material

Supplemental Materials

SUPPORTING INFORMATION

Additional supporting information can be found online in the Supporting Information section at the end of this article.

## Figures and Tables

**FIGURE 1:**
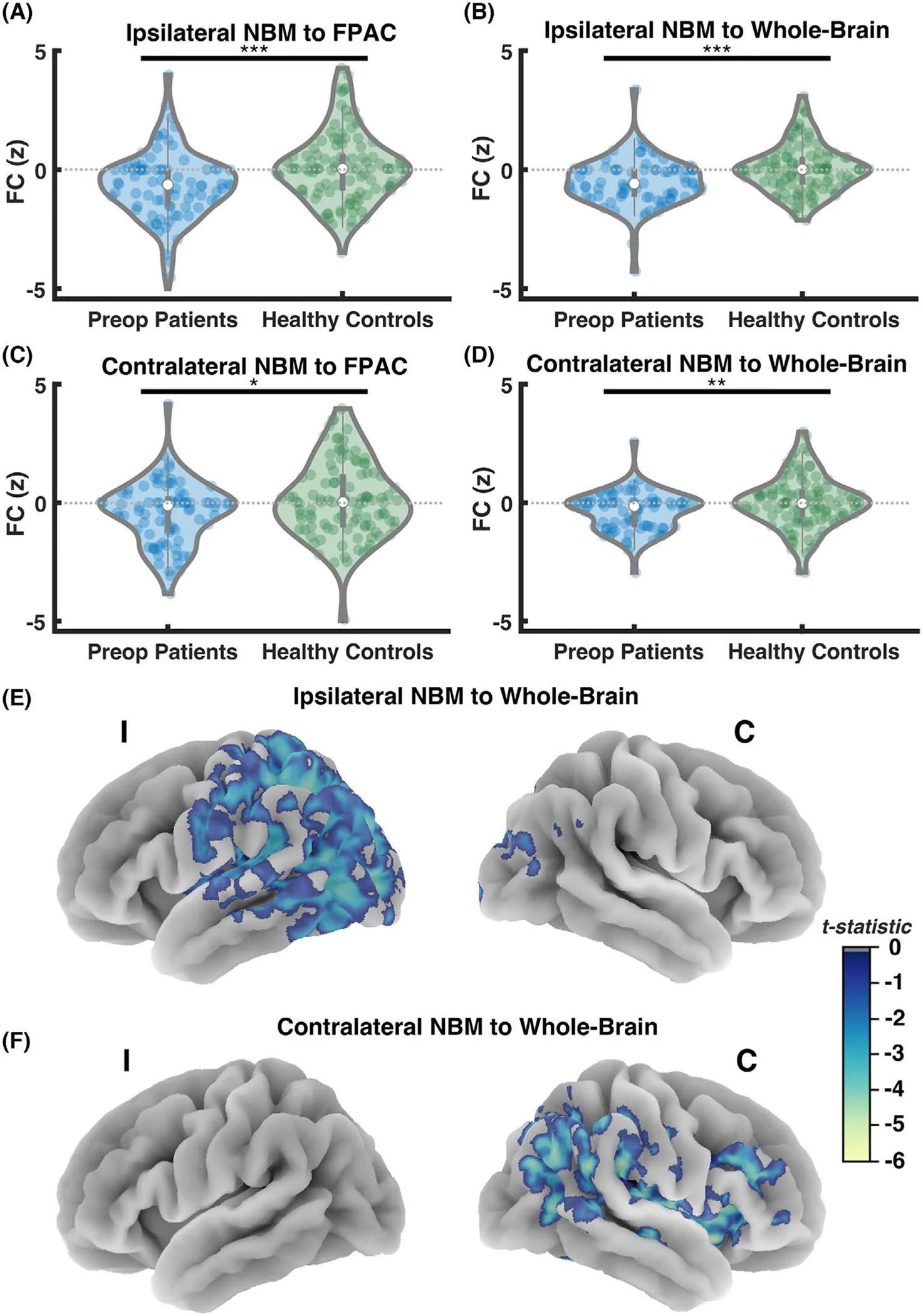
Nucleus basalis of Meynert (NBM) exhibits reduced connectivity in patients with temporal lobe epilepsy compared to controls. Violin plots show Fisher *z*-transformed Pearson correlations between ipsilateral NBM node time series (A, B) and the contralateral node time series (C, D) with the frontoparietal association cortex (FPAC) as well as all other nodes in the brain (whole-brain). Data include 75 patients with temporal lobe epilepsy and 106 healthy controls. Voxelwise connectivity maps show threshold-free cluster enhancement (TFCE)-corrected *t*-statistics, illustrating reductions in ipsilateral (E) and contralateral (F) NBM connectivity in preoperative patients compared to controls. Negative contrasts (patients < controls) are shown, and no positive differences (patients > controls) survived correction. One patient in panel A had functional connectivity (FC) = −7.60, which falls outside the plotted axis range and thus was not shown for visualization purposes. Group differences were assessed using independent samples *t*-tests with false discovery rate correction. *p < .05, **p < .01, ***p < .001. C, contralateral; I, ipsilateral.

**FIGURE 2:**
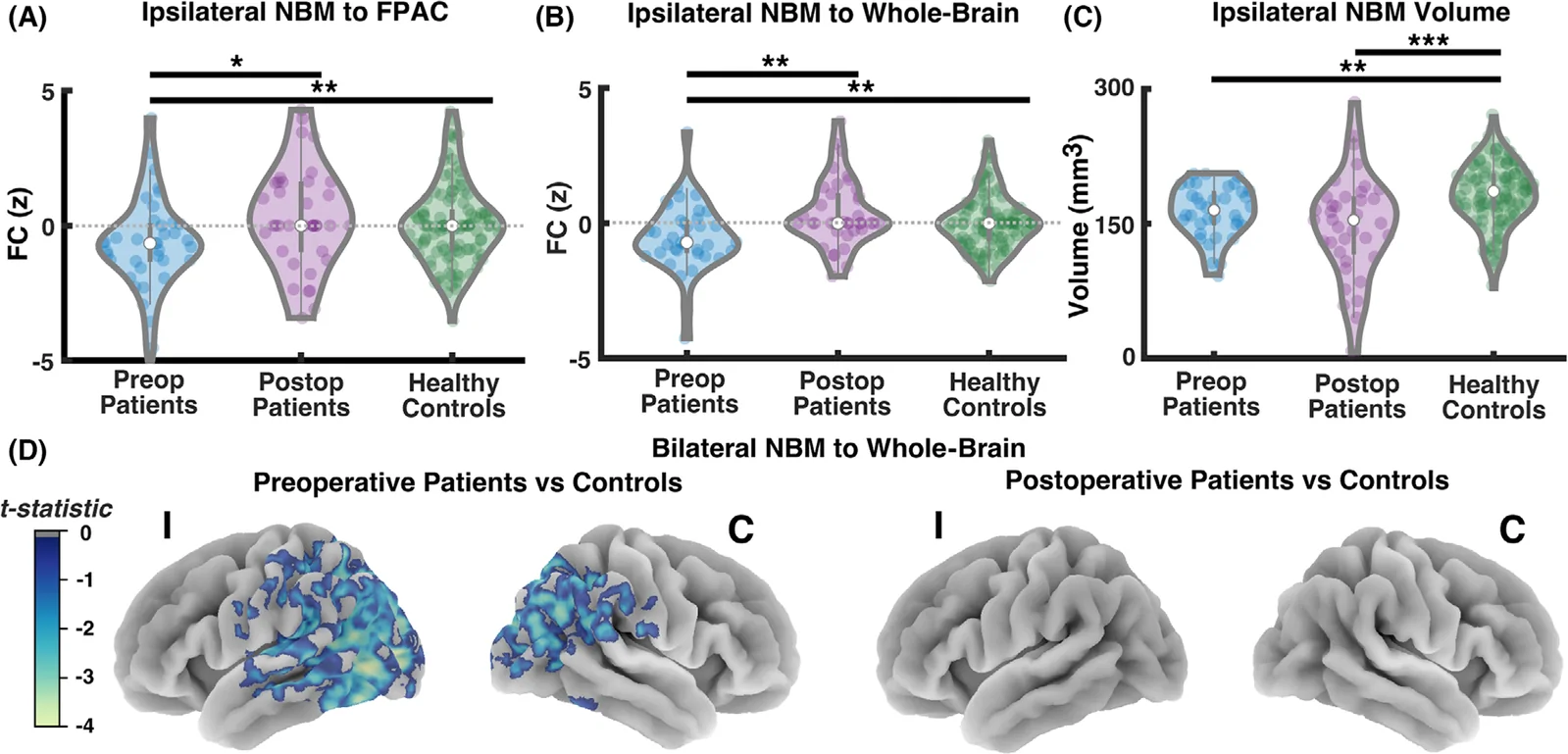
Nucleus basalis of Meynert (NBM) functional connectivity increases following surgery. Violin plots show Fisher *z*-transformed functional connectivity between NBM node time series and (A) frontoparietal association cortex (FPAC) and (B) other nodes in the brain (whole-brain). Both ipsilateral and contralateral NBM demonstrated increased connectivity following surgery, such that postoperative connectivity no longer differed from controls. (C) Patients exhibited reduced ipsilateral NBM volume relative to controls both before and after surgery. (D) Voxelwise connectivity maps show threshold-free cluster enhancement-corrected *t*-statistics, illustrating widespread reductions in bilateral NBM connectivity in preoperative patients compared to controls, which were not observed postoperatively. Negative contrasts are shown (patients < controls), and no positive differences (patients > controls) survived correction. Data include 34 patients with temporal lobe epilepsy and 106 controls. Within-patient changes were assessed using paired *t*-tests; patient–control comparisons used independent samples *t*-tests. All *p*-values were false discovery rate-corrected. One patient in panel A had functional connectivity (FC) = −7.60, which falls outside the plotted axis range and thus was not shown for visualization purposes. *p < .05, **p < .01, ***p < .001. C, contralateral; I, ipsilateral.

**FIGURE 3:**
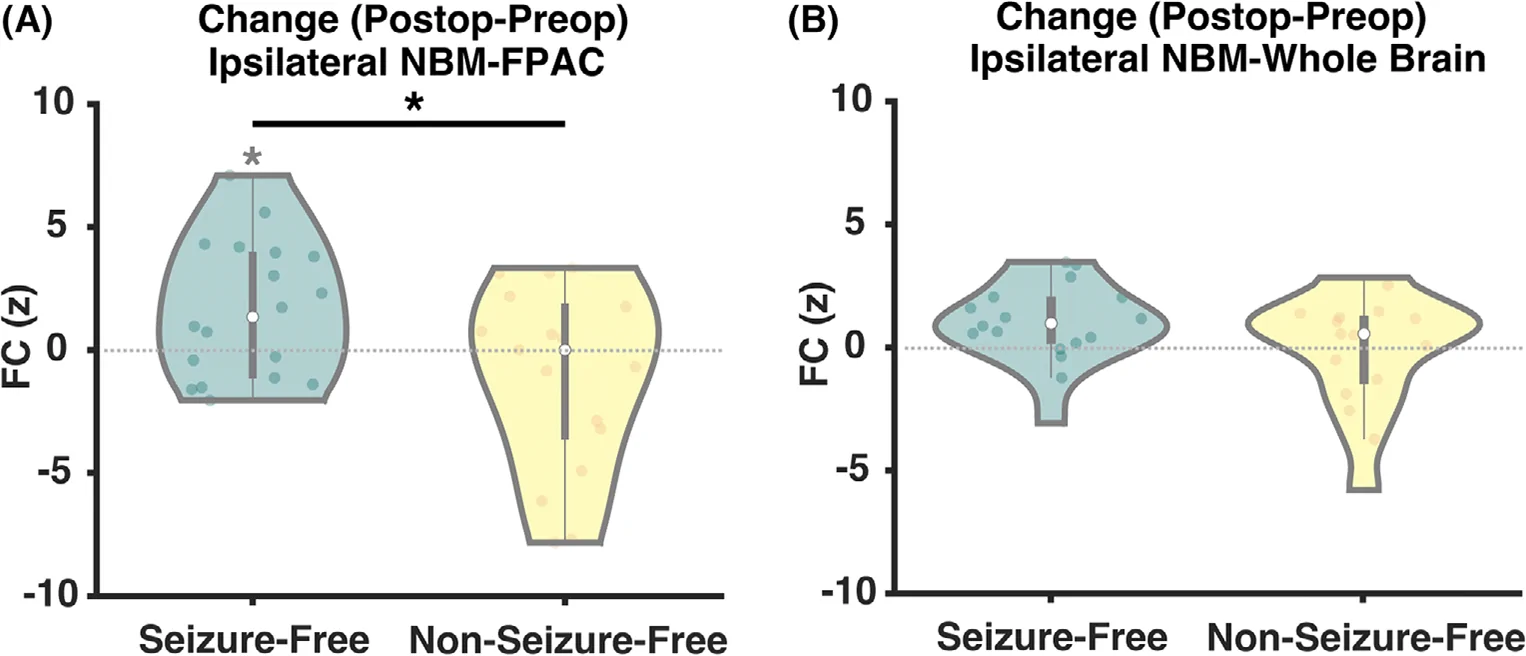
Seizure-free patients show increased change in ipsilateral nucleus basalis of Meynert (NBM)–frontoparietal association cortex (FPAC) functional connectivity (FC) compared to non-seizure-free patients. Violin plots show change in Fisher *z*-transformed functional connectivity between the ipsilateral NBM and FPAC, calculated as postoperative minus preoperative connectivity for each patient. Stars above individual violins indicate results of one-sample *t*-tests testing whether the mean change differed significantly from zero (dashed line). Patients who achieved seizure freedom (Engel IA, *n* = 18) demonstrated greater increases in NBM-FPAC connectivity compared with non-seizure-free patients (non-Engel IA, *n* = 16). Data include 34 patients with temporal lobe epilepsy. Group differences were assessed using independent samples *t*-tests and false discovery rate-corrected. *p < .05.

**FIGURE 4:**
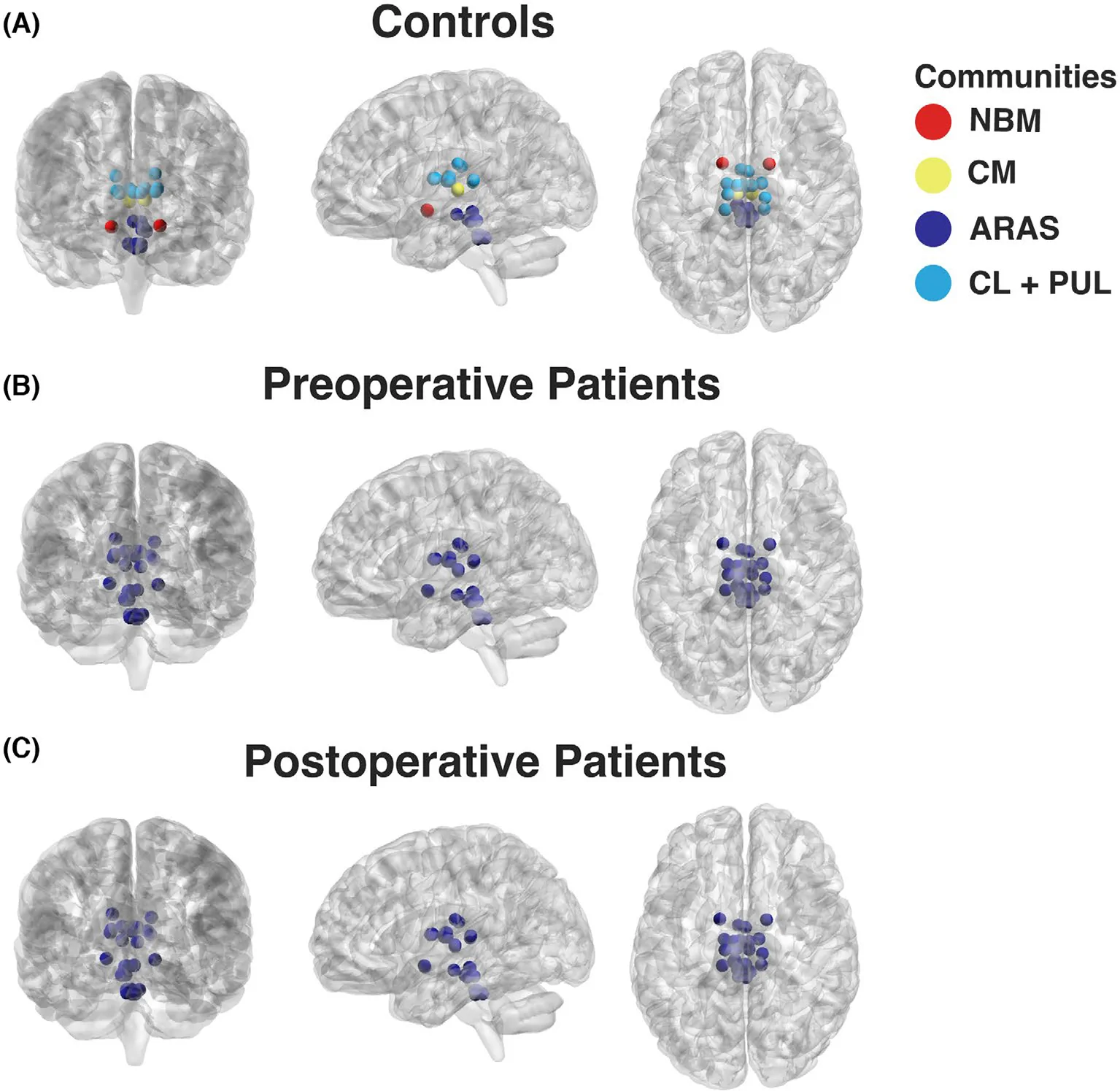
Disrupted subcortical network community structure in patients with temporal lobe epilepsy (TLE) persists after surgery. Louvain community detection was applied to individual connectivity matrices and used to identify group-level network communities. Community assignments of subcortical arousal regions of interest were compared across controls, preoperative patients, and postoperative patients to assess reorganization or preservation of subcortical network topology across disease and surgical cohorts. Community detection revealed reduced segregation (decreased number of communities) of arousal-related subcortical structures in preoperative patients with TLE compared to controls, and this network architecture persisted in patients following surgery. Data include 34 patients with unilateral TLE before and after surgery and 106 controls. ARAS, ascending reticular activating system; CL, central lateral nucleus; CM, centromedian nucleus; NBM, nucleus basalis of Meynert; PUL, pulvinar nucleus.

**TABLE 1:** Demographics and disease characteristics.

Characteristic	Patients, *n* = 75	Controls, *n* = 106	*p*
Age, years	40.3 ± 12.3	37.3 ± 13.1	.18
Sex, female	37 (49.3%)	56 (52.8%)	.95
Handedness, right	61 (81.3%)	91 (85.8%)	.63
Epilepsy duration, years	18.5 ± 14.1		
Seizure frequency, monthly			
FPC	6.5 ± 25.3		
FIC	7.5 ± 11.5		
FBTC	.9 ± 2.3		
History of FBTC, yes	44 (58.7%)		
Epileptogenic side			
Right	40 (53.3%)		
Left	35 (46.7%)		
Number of ASMs	2.3 ± .73		
Surgery Cohort, *n* = 34			
Surgery			
Selective amygdalohippocampectomy	24 (70.6%)		
Anterior temporal lobectomy	9 (26.5%)		
Laser ablation	1 (2.9%)		
Previous surgery, yes	3 (8.8%)		
Time to postoperative scan, months	30.3 ± 15.1		
Pathology			
Mesial temporal sclerosis	26 (76.5%)		
Gliosis	6 (17.6%)		
Normal	3 (8.8%)		

*Note:* For continuous variables, data are shown as mean ± SD, and statistical testing is performed with an independent samples *t*-test or Mann-Whitney *U*-test for parametric and nonparametric data determined using the Anderson-Darling test, respectively. For categorical variables, data are shown as count (percentage), and statistical testing is performed with a chi-squared test. Seizure type and frequency were determined from the clinical visit most recent to the preoperative functional magnetic resonance imaging scan. Comparison of clinical variables across seizure-free and non-seizure-free patients of the surgery cohort is reported in Table S1, and across preoperative and postoperative cohorts in Table S2.

Abbreviations: ASM, antiseizure medication; FBTC, focal to bilateral tonic-clonic seizures; FIC, focal impaired consciousness seizures; FPC, focal preserved consciousness seizures.

## Data Availability

Data may be made available upon reasonable request.

## Key points

- Preoperative bilateral NBM functional connectivity is reduced in patients with temporal lobe epilepsy.
- Seizure-free patients show greater recovery of ipsilateral NBM functional connectivity than non-seizure-free patients.
- NBM functional connectivity changes in seizure-free patients were not explained by time to postoperative scan nor changes in NBM volume.
