# Supplementary material for: Nucleus basalis functional connectivity aberrations in temporal lobe epilepsy and improvements after successful epilepsy surgery
Source: Epilepsia. 2026 Aug 19;67(11):5919–31. doi: 10.1002/epi.70451 (PMC13650722; doi:10.1002/epi.70451)
Supplement: Supplementary file 1 [file EPI-67-5919-s001.docx]

| **Supplementary Table 1. Demographics and Disease Characteristics Across Seizure Outcomes** | | | |
| --- | --- | --- | --- |
|  | **Seizure-free (n=18)** | **Non-seizure-free (n=16)** | **p-Value** |
| **Age, *years*** | 43.3 ± 13.1 | 38.2 ± 8.8 | 0.19 |
| **Sex, *female*** | 9 (50.0%) | 7 (43.8%) | 0.72 |
| **Handedness, *right*** | 14 (77.8%) | 13 (81.2%) | 0.80 |
| **Epilepsy duration, *years*** | 22.6 ± 15.4 | 21.6 ± 15.8 | 0.86 |
| **Seizure frequency, *monthly*** |  |  |  |
| **FPC** | 3.2 ± 5.8 | 4.9 ± 14.8 | 0.66 |
| **FIC** | 4.7 ± 7.0 | 6.0 ± 6.8 | 0.59 |
| **FBTC** | 0.2 ± 0.6 | 1.3 ± 2.3 | 0.06 |
| **History of FBTC, *yes*** | 8 (47.1%) | 11 (68.8%) |  |
| **Epileptogenic side** |  |  | 0.80 |
| **Right** | 13 (72.2%) | 12 (75.0%) |  |
| **Left** | 5 (27.8%) | 4 (25.0%) |  |
| **Number of ASMs** | 2 ± 0.53 | 2 ± 0.60 | 0.14 |
| **Surgery** |  |  | 0.56 |
| **Selective amygdalohippocampectomy** | 13 (72.2%) | 11 (68.8%) |  |
| **Anterior temporal lobectomy** | 5 (27.8%) | 4 (25.0%) |  |
| **Laser ablation** | 0 (0%) | 1 (6.2%) |  |
| **Previous surgery, *yes*** | 1 (5.6%) | 2 (12.5%) | 0.48 |
| **Time to postoperative scan, *months*** | 33.6 ± 17.2 | 27.7 ± 11.9 | 0.26 |
| **Pathology** |  |  | 0.37 |
| **Mesial temporal sclerosis** | 15 (88.2%) | 11 (64.7%) |  |
| **Gliosis** | 2 (11.8%) | 4 (23.5%) |  |
| **Normal** | 0 (0%) | 2 (11.8%) |  |

**Supplementary Table 1. Demographics and Disease Characteristics Across Seizure Outcomes.** For continuous variables, data are shown mean ± standard deviation, and statistical testing is performed with an independent samples t-test or Mann-Whitney U test for parametric and non-parametric data, respectively, determined using the Anderson Darling test. For categorical variables, data are shown count (percentage), and statistical testing is performed with a chi-squared test. Seizure type and frequency were determined from clinical visit most recent to preoperative fMRI scan. Abbreviations: FPC = focal preserved consciousness seizures; FIC = focal impaired-consciousness seizures; FBTC = focal to bilateral tonic-clonic seizures; ASMs = anti-seizure medications.

| **Supplementary Table 2. Demographics and Disease Characteristics Across Preoperative and Postoperative Cohorts** | | | |
| --- | --- | --- | --- |
|  | **Preoperative (n=75)** | **Postoperative (n=34)** | **p-Value** |
| **Age, *years*** | 40.28 ± 12.31 | 40.91 ± 11.41 | 0.80 |
| **Sex, *female*** | 37 (49.3%) | 16 (47.1%) | 0.83 |
| **Handedness, *right*** | 61 (81.3%) | 27 (79.4%) | 0.81 |
| **Epilepsy duration, *years*** | 18.5 ± 14.1 | 22.1 ± 15.4 | 0.28 |
| **Seizure frequency, *monthly*** |  |  |  |
| **FPC** | 6.5 ± 25.3 | 4.0 ± 10.9 | 0.63 |
| **FIC** | 7.5 ± 11.5 | 5.3 ± 6.9 | 0.50 |
| **FBTC** | 0.9 ± 2.3 | 0.7 ± 1.7 | 0.59 |
| **History of FBTC, *yes*** | 44 (58.7%) | 19 (55.9%) | 0.78 |
| **Epileptogenic side** |  |  | 0.05 |
| **Right** | 40 (53.3%) | 25 (73.5%) |  |
| **Left** | 35 (46.7%) | 9 (26.5%) |  |
| **Number of ASMs** | 2.3 ± 0.73 | 2.0 ± 0.58 | 0.09 |

**Supplementary Table 2. Demographics and Disease Characteristics Across Preoperative and Postoperative Cohorts.** For continuous variables, data are shown mean ± standard deviation, and statistical testing is performed with an independent samples t-test or Mann-Whitney U test for parametric and non-parametric data, respectively, determined using the Anderson Darling test. For categorical variables, data are shown count (percentage), and statistical testing is performed with a chi-squared test. Seizure type and frequency were determined from clinical visit most recent to preoperative fMRI scan. Note that all postoperative patients are included in the initial preoperative cohort. Abbreviations: FPC = focal preserved consciousness seizures; FIC = focal impaired-consciousness seizures; FBTC = focal to bilateral tonic-clonic seizures; ASMs = anti-seizure medications.

| Supplementary Table 3. Complete List of Atlas Regions | | |
| --- | --- | --- |
| **Atlas Region Name** | | |
| Banks of the Superior Temporal Sulcus^#^ | Pars orbitalis^#^ | Hippocampus^+^ |
| Caudal Anterior Cingulate Cortex^#^ | Pars triangularis^#^ | Pallidum^+^ |
| Caudal Middle Frontal Cortex^#^ | Pericalcarine cortex^#^ | Putamen^+^ |
| Cuneus^#^ | Postcentral gyrus^#^ | NBM^△^ |
| Entorhinal Cortex^#^ | Posterior cingulate cortex^#^ | Cuneiform/subcuneiform^†^ |
| Frontal pole^#^ | Precentral gyrus^#^ | DR*^†^ |
| Fusiform Area^#^ | Precuneus^#^ | Locus coerulus^†^ |
| Inferior Parietal Cortex^#^ | Rostral anterior cingulate cortex^#^ | MR*^†^ |
| Inferior Temporal Cortex^#^ | Rostral middle frontal cortex^#^ | Parabrachial complex^†^ |
| Insula^#^ | Superior frontal cortex^#^ | Pontis oralis^†^ |
| Isthmus of Cingulate Cortex^#^ | Superior parietal cortex^#^ | Pedunculopontine nucleus^†^ |
| Lateral occipital cortex^#^ | Superior temporal cortex^#^ | Ventral tegmental area*^†^ |
| Lateral orbitofrontal cortex^#^ | Supramarginal gyrus^#^ | Anterior nucleus^‡^ |
| Lingual gyrus^#^ | Temporal pole^#^ | Medial nucleus^‡^ |
| Medial orbitofrontal cortex^#^ | Transverse temporal gyrus^#^ | Centre median^‡^ |
| Middle temporal cortex^#^ | Amygdala^+^ | Lateral dorsal/lateral posterior^‡^ |
| Parahippocampal gyrus^#^ | Accumbens^+^ | Ventral thalamus^‡^ |
| Paracentral gyrus^#^ | Caudate | Pulvinar^‡^ |
| Pars opercularis^#^ | Cerebellum | Central lateral^‡^ |

**Supplementary Table 3. Complete list of atlas regions.** ^+^Harvard Oxford Atlas^35^; ^#^Desikan-Killany Atlas Cortical Regions^36^; ^△^Nucleus Basalis of Meynert Segmentation^27^; ^†^Harvard Ascending Arousal Network Atlas^37^; ^‡^Thalamic Nuclei Segmentation^30^; *Midline regions.
