# Supplementary material for: Nucleus basalis functional connectivity aberrations in temporal lobe epilepsy and improvements after successful epilepsy surgery
Source: Epilepsia. Author manuscript; Available in PMC 2026 Oct 9. (PMC13650722; doi:10.1002/epi.70451)
Supplement: Supplementary file 1 [file NIHMS2209142-supplement-Supplemental_Materials.pdf]

Supplementary Table 1. Demographics and Disease Characteristics Across Seizure Outcomes			
	**Seizure-free (n=18)**	**Non-seizure-free (n=16)**	**p-Value**
**Age, *years***	43.3 ± 13.1	38.2 ± 8.8	0.19
**Sex, *female***	9 (50.0%)	7 (43.8%)	0.72
**Handedness, *right***	14 (77.8%)	13 (81.2%)	0.80
**Epilepsy duration, *years***	22.6 ± 15.4	21.6 ± 15.8	0.86
**Seizure frequency, *monthly***			
**FPC**	3.2 ± 5.8	4.9 ± 14.8	0.66
**FIC**	4.7 ± 7.0	6.0 ± 6.8	0.59
**FBTC**	0.2 ± 0.6	1.3 ± 2.3	0.06
**History of FBTC, *yes***	8 (47.1%)	11 (68.8%)	
**Epileptogenic side**			0.80
**Right**	13 (72.2%)	12 (75.0%)	
**Left**	5 (27.8%)	4 (25.0%)	
**Number of ASMs**	2 ± 0.53	2 ± 0.60	0.14
**Surgery**			0.56
**Selective amygdalohippocampectomy**	13 (72.2%)	11 (68.8%)	
**Anterior temporal lobectomy**	5 (27.8%)	4 (25.0%)	
**Laser ablation**	0 (0%)	1 (6.2%)	
**Previous surgery, *yes***	1 (5.6%)	2 (12.5%)	0.48
**Time to postoperative scan, *months***	33.6 ± 17.2	27.7 ± 11.9	0.26
**Pathology**			0.37
**Mesial temporal sclerosis**	15 (88.2%)	11 (64.7%)	
**Gliosis**	2 (11.8%)	4 (23.5%)	
**Normal**	0 (0%)	2 (11.8%)	

Supplementary Table 1. Demographics and Disease Characteristics Across Seizure Outcomes. For continuous variables, data are shown mean ± standard deviation, and statistical testing is performed with an independent samples t-test or Mann-Whitney U test for parametric and non-parametric data, respectively, determined using the Anderson Darling test. For categorical variables, data are shown count (percentage), and statistical testing is performed with a chi-squared test. Seizure type and frequency were determined from clinical visit most recent to preoperative fMRI scan. Abbreviations: FPC = focal preserved consciousness seizures; FIC = focal impaired-consciousness seizures; FBTC = focal to bilateral tonic-clonic seizures; ASMs = anti-seizure medications.

**Supplementary Table 2. Demographics and Disease Characteristics Across Preoperative and Postoperative Cohorts**			
	**Preoperative (n=75)**	**Postoperative (n=34)**	**p-Value**
**Age, *years***	40.28 ± 12.31	40.91 ± 11.41	0.80
**Sex, *female***	37 (49.3%)	16 (47.1%)	0.83
**Handedness, *right***	61 (81.3%)	27 (79.4%)	0.81
**Epilepsy duration, *years***	18.5 ± 14.1	22.1 ± 15.4	0.28
**Seizure frequency, *monthly***			
**FPC**	6.5 ± 25.3	4.0 ± 10.9	0.63
**FIC**	7.5 ± 11.5	5.3 ± 6.9	0.50
**FBTC**	0.9 ± 2.3	0.7 ± 1.7	0.59
**History of FBTC, *yes***	44 (58.7%)	19 (55.9%)	0.78
**Epileptogenic side**			0.05
**Right**	40 (53.3%)	25 (73.5%)	
**Left**	35 (46.7%)	9 (26.5%)	
**Number of ASMs**	2.3 ± 0.73	2.0 ± 0.58	0.09

Supplementary Table 2. Demographics and Disease Characteristics Across Preoperative and Postoperative Cohorts. For continuous variables, data are shown mean ± standard deviation, and statistical testing is performed with an independent samples t-test or Mann-Whitney U test for parametric and non-parametric data, respectively, determined using the Anderson Darling test. For categorical variables, data are shown count (percentage), and statistical testing is performed with a chi-squared test. Seizure type and frequency were determined from clinical visit most recent to preoperative fMRI scan. Note that all postoperative patients are included in the initial preoperative cohort. Abbreviations: FPC = focal preserved consciousness seizures; FIC = focal impaired-consciousness seizures; FBTC = focal to bilateral tonic-clonic seizures; ASMs = anti-seizure medications.

Supplementary Table 3. Complete List of Atlas Regions		
**Atlas Region Name**		
Banks of the Superior Temporal Sulcus#	Pars orbitalis#	Hippocampus+
Caudal Anterior Cingulate Cortex#	Pars triangularis#	Pallidum+
Caudal Middle Frontal Cortex#	Pericalcarine cortex#	Putamen+
Cuneus#	Postcentral gyrus#	NBM△
Entorhinal Cortex#	Posterior cingulate cortex#	Cuneiform/subcuneiform†
Frontal pole#	Precentral gyrus#	DR*†
Fusiform Area#	Precuneus#	Locus coerulus†
Inferior Parietal Cortex#	Rostral anterior cingulate cortex#	MR*†
Inferior Temporal Cortex#	Rostral middle frontal cortex#	Parabrachial complex†
Insula#	Superior frontal cortex#	Pontis oralis†
Isthmus of Cingulate Cortex#	Superior parietal cortex#	Pedunculopontine nucleus†
Lateral occipital cortex#	Superior temporal cortex#	Ventral tegmental area*†
Lateral orbitofrontal cortex#	Supramarginal gyrus#	Anterior nucleus‡
Lingual gyrus#	Temporal pole#	Medial nucleus‡
Medial orbitofrontal cortex#	Transverse temporal gyrus#	Centre median‡
Middle temporal cortex#	Amygdala+	Lateral dorsal/lateral posterior‡
Parahippocampal gyrus#	Accumbens+	Ventral thalamus‡
Paracentral gyrus#	Caudate	Pulvinar‡
Pars opercularis#	Cerebellum	Central lateral‡

Supplementary Table 3. Complete list of atlas regions. +Harvard Oxford Atlas[35]; #Desikan-Killany Atlas Cortical Regions[36]; △Nucleus Basalis of Meynert Segmentation[27]; †Harvard Ascending Arousal Network Atlas[37]; ‡Thalamic Nuclei Segmentation[30]; *Midline regions.
